# HDL protein composition differs between young white European and South Asian men before and after weight gain

**DOI:** 10.1042/CS20258040

**Published:** 2025-12-18

**Authors:** Jack D. Beazer, James McLaren, Christina Christoffersen, Maria J. Ferraz, Monique T. Mulder, Delyth Graham, Helen Karlsson, Stefan A. Ljunggren, Jason M.R. Gill, Dilys J. Freeman

**Affiliations:** 1 School of Cardiovascular and Metabolic Health, Glasgow Cardiovascular Research Centre, University of Glasgow, 126 University Place, Glasgow, G12 8TA, U.K.; 2 Department of Clinical Biochemistry, Section 3-01-3, Rigshospitalet, Copenhagen, 2100, Denmark; 3 Institute of Biomedical Sciences, University of Copenhagen, Copenhagen, 2200, Denmark; 4 Medical Biochemistry, Leiden Institute of Chemistry, Gorlaeus Building, Leiden, 2333 CC, Netherlands; 5 Division of Pharmacology, Vascular and Metabolic Diseases, Department of Internal Medicine, Erasmus University Medical Center, Rotterdam, 3015 GD, Netherlands; 6 Occupational and Environmental Medicine Center in Linköping, and Department of Health, Medicine and Caring Sciences, Linköping University, Linköping, SE-58183, Sweden

**Keywords:** apolipoproteins, high-density lipoprotein, lipoproteins, obesity, South Asians

## Abstract

South Asians (SAs) in the UK are at an increased risk of cardiovascular disease (CVD), develop type 2 diabetes mellitus at a lower age and body mass index, and have a lower high-density lipoprotein cholesterol (HDL-C) concentration than their white European (EU) counterparts. The failure of HDL-C raising therapies for CVD risk reduction has turned attention to its composition and function. A previous study comparing the effect of moderate weight gain on SA and EU men found baseline and weight gain-induced ethnic differences in body composition, adipocyte function and insulin resistance (ClinicalTrials.gov registration: NCT02399423). This study investigated differences in HDL protein composition, subclass distribution and *in vitro* vascular functions at baseline and after weight gain in the same cohort of men. HDL protein composition was determined by nano liquid chromatography tandem mass spectrometry using label-free quantification. HDL subclass distribution was measured by native gel electrophoresis. HDL *in vitro* paraoxonase-1 (PON-1) activity was measured by monitoring the PON-1 mediated hydrolysis of phenylacetate. *In vitro* HDL anti-inflammatory function was assessed in an endothelial cell assay of adhesion molecule inhibition. SAs had higher levels of immunity- and inflammation-related proteins and a detrimental profile of lipid metabolism-related proteins at baseline and with weight gain (including lower apolipoprotein (apo) A-IV and apoF and higher apoC-III) compared with EU. HDL subclass distribution and *in vitro* vascular function were not different between EUs and SAs. HDL protein composition reflects systemic physiology and acts as a mechanistic marker of impaired lipid metabolism in SAs.

## Introduction

There has recently been a notable acceleration in the incidence of obesity in parts of the world where undernutrition was typically a greater concern, including in South Asia (particularly India, Pakistan, Bangladesh and Sri Lanka) [1]. For example, the proportion of obese adults in India increased by 6.4 percentage points in men and 5.1 percentage points in women between 2015 and 2021, with overweight or obese men and women now accounting for 44.0% and 41.2% of India’s population, respectively [2]. South Asians (SAs) living in the UK have a significantly increased risk of cardiovascular diseases (CVDs) and develop type 2 diabetes mellitus (T2DM) over a decade earlier than their white European (EU) counterparts [3,4]. A prevalence of T2DM equivalent to that of the EUs at a body mass index (BMI) of 30 kg/m^2^ was observed at a BMI of 21.6 kg/m^2^ in SAs [5], and T2DM is a stronger risk factor for all-cause mortality in SAs [6]. There are ethnic differences in white adipose tissue distribution which may contribute to the different pattern of cardiometabolic risk; a meta-analysis of 11 studies comparing SAs to EUs found that, at a lower BMI, SAs have higher subcutaneous adipose tissue and ectopic liver fat, but no difference in visceral adipose tissue (VAT), perhaps demonstrating an impaired ability of SAs to expand their VAT [7].

Lipoprotein metabolism and its dysregulation are well established at the intersection of cardiovascular and metabolic disease [8,9], with high-density lipoprotein cholesterol (HDL-C) inversely associated with CVD risk. However, the failure of HDL-raising pharmacological interventions to reduce CVD risk has turned attention to its composition and vascular functions. We have previously corroborated reports of detrimental HDL composition and reduced function in men with impaired glucose regulation compared with healthy controls. The same study also found that HDL of endurance athletes was not functionally superior to controls, despite a favourable composition, in a cross-sectional study of middle-aged EU men [10]. Though SAs are known to have lower HDL-C [11], studies into SA HDL composition and function are limited. Two cross-sectional studies have been performed in the Netherlands: one in healthy neonates, adolescents and adults [12] and another in participants with T2DM [13]. Both found limited differences in HDL composition and function between SAs and EUs. Bakker et al. observed lower antioxidant capacity in adult SA HDL [12], while Yuan et al. saw lower apolipoproteins (apo) A-I and apoA-II and phospholipid content in small HDL, but higher apoA-I, apoA-II, phospholipid and cholesterol content in large HDL of SAs with T2DM. Bakker et al. also investigated the impact of five- and eight-day high- and low-calorie diets on HDL function. The eight-day low-calorie diet reduced the anti-inflammatory ability of HDL in adult SAs with no change in EU HDL [12]. However, these studies were unable to reveal how HDL may change in the same individual with changes in body composition or metabolic physiology, especially given that lipoprotein metabolism is not static, while the short dietary interventions may not have been sufficient to provoke sustained, detectable changes in the aspects of HDL composition measured.

A recent human intervention study in young, healthy SA and EU men living in Glasgow, Scotland, U.K, investigated the effect of 5–7% weight gain over 4–6 weeks on body composition and adiposity, the metabolic response to a standardised test meal and adipocyte size and volume [14]. McLaren et al. reported significantly higher postprandial insulin and triglyceride concentrations in SAs at baseline, while weight gain resulted in substantial increases in SA fasting and postprandial insulin concentrations. The present study therefore aimed to establish the effect of weight gain, which changed body composition and increased insulin resistance, on HDL composition and function in the same cohort of men. Specifically, this study aimed to investigate how HDL (i) constituents, (ii) subclass distribution, (iii) protein composition and (iv) *in vitro* paraoxonase-1 (PON-1) activity and vascular anti-inflammatory functions differ between SAs and EUs at baseline and after sustained weight gain in the same individuals.

## Methods

### Participant recruitment

This cohort was originally collected as part of the ‘Glasgow visceral & ectopic fat with weight gain in South Asians (GlasVEGAS)’ study, performed at the University of Glasgow between 2015 and 2017 and funded by the European Medical Information Framework (project number 60315/1, ClinicalTrials.gov identifier: NCT02399423). This study was carried out in accordance with the World Medical Association Declaration of Helsinki, and all subjects provided written informed consent at the time of recruitment. The major findings of this study are described by McLaren et al. [14]. The study set out to compare the physiological and metabolic changes associated with weight gain in young, lean SA and EU men. Participants were asked to gain ~7% of their baseline body weight over 4–6 weeks by eating until fuller than usual and increasing intake of high-sugar snacks. Before each study visit, participants were provided with all of their food based on measured energy expenditure in order to maintain their weight for three days prior to metabolic and anthropometric measurements. At each study visit, participants gave a fasted blood sample before consuming a standard mixed test meal (containing ~ 800 kcal, 37% fat, 47% carbohydrate, 17% protein) and underwent a mixed-meal tolerance test. Whole-body MRI scans were taken to measure changes in subcutaneous, visceral and liver fat distribution with weight gain. Participant fitness at each study visit was also measured with a continuous incremental uphill walking treadmill test. The study was powered to detect a one standard deviation difference in postprandial glucose and insulin concentrations with 80.3% power in *n*=21 EUs and *n*=14 SAs at baseline and weight gain [14].

### Isolation of HDL from plasma

HDL was isolated from plasma by sequential density sodium bromide ultracentrifugation using a Beckman Coulter TLA 120.2 rotor in an Optima MAX TL ultracentrifuge, as described previously [10]. Very low-density lipoprotein (VLDL) and low-density lipoprotein (LDL) were first removed from plasma by flotation at 1.063 g/ml (100,000 rpm / 358,400 x g average for 2.5 hours at 23°C) before HDL was isolated at a density of 1.21 g/ml (100,000 rpm / 358,400 x g average for 5 hours at 23°C). HDL fractions were stored at -80°C.

### Measurement of HDL constituents

HDL apoA-I and serum amyloid A1 (SAA-1) were measured by R&D Systems DuoSet ELISA (#DY3664 and #DY3019) according to the manufacturer’s protocol. HDL cholesterol content was measured by cholesterol quantitation kit (#MAK043, Sigma-Aldrich). ApoM content of HDL was measured using a custom enzyme-linked immunosorbent assay (ELISA) as detailed in [15]. HDL sphingosine-1-phosphate (S1P) content was measured by liquid chromatography tandem mass spectrometry (LC-MS/MS) with ^13^C_5_-S1P as the internal standard, as previously described [16].

### Measurement of HDL subclass distribution

Native gel electrophoresis was used to semi-quantitatively measure HDL subclass distribution as previously described [10]. After staining the gels with QC Colloidal Coomassie stain (Bio-Rad Laboratories #1610803) and imaging on a Licor FC Odyssey scanner, standardised retention factors (RF) were calculated for each protein standard band (NativeMark, Thermo Fisher Scientific #LC0725), and a linear standard curve was drawn, from which the maximum and minimum RF values for each of the following HDL subclasses were calculated based on their diameter: 2b 12.9–9.7 nm, 2a 9.7–8.8 nm, 3a 8.8–8.2 nm, 3b 8.2–7.8 nm, 3c 7.8–7.2 nm [17]. Summed HDL 2 and HDL 3 were calculated by summing the relevant subfraction signals. The signal for each subclass was expressed as a percentage of the total HDL signal. Size-based separation of HDL subclasses using gel electrophoresis has been shown to correlate well with density-based methods [18].

### Proteomic analysis of HDL

Proteomic analysis of HDL was performed using nano LC-MS/MS (nLC-MS/MS) as described elsewhere [10]. In brief, 10 μg of HDL proteins were reduced with dithiothreitol, alkylated with iodoacetamide and digested with trypsin. Peptides were cleaned using C18 ZipTips (Merck Millipore) and lyophilised before reconstitution in 0.1% formic acid. Concentration was determined using a Nanodrop 1000 (Thermo Fisher Scientific). Peptides (200 ng) were separated on a 20-cm EASY-Spray C18 connected to an EASY-nLC 1200 (Thermo Fisher Scientific, Waltham, MA, U.S.A.) using a linear gradient of 0.1% formic acid in water (A) and 0.1% formic acid in 80% acetonitrile (B) (7–40% B over 75 minutes followed by 40–100% B over 13 minutes and 2 minutes of holding at 100% B). Automated online analyses were performed with a QExactive High Field mass spectrometer (Thermo Fisher Scientific) with a nano-electrospray source and a top10 data-dependent method. Proteins with at least two peptides, of which one was unique and identified in at least 50% of the HDL samples in a given group, were included in further analysis. Label-free quantification (LFQ) was performed using the built-in LFQ algorithm. All samples were analysed for protein composition with the exception of four SA samples due to sample volume constraints, resulting in *n*=11 at baseline and *n*=13 with weight gain in this ethnicity.

### HDL paraoxonase-1 activity assay

The rate of conversion of phenyl acetate to phenol and acetate mediated by HDL PON-1 was measured using a kinetic spectrophotometric technique described previously [19].

### HDL anti-inflammatory function assay

The *in vitro* anti-inflammatory function of HDL was assessed using an endothelial cell model of vascular inflammation described previously [10]. Human microvascular endothelial cells (ATCC-CRL-3243, LGC Standards, Middlesex, U.K) were preincubated with HDL (based on 300 μg/ml apoA-I) for 4 hours before the addition of 5 ng/ml tumour necrosis factor alpha (TNFα) for 24 hours. Cells were lysed, and western blotting was performed to detect vascular cell adhesion molecule-1 (VCAM-1) expression. Anti-inflammatory function was expressed as percentage inhibition of VCAM-1 expression per µg HDL total protein.

### Statistical analysis

As an interventional repeated measures study, the same individuals were followed with weight gain. Mixed effects models (MEMs) were used to compare HDL constituents, subclass distribution and *in vitro* vascular functions, including participant identifier as a random effect [20]. MEMs were deemed valid if there was a normal distribution of residuals. Where residuals were not normally distributed, the input data were log-transformed. *Post hoc* pairwise comparisons were made with Tukey tests. Statistical significance was assumed at *P*<0.05 for all comparisons and reported as p_ETHNICITY_, p _WEIGHT GAIN_ and p_INTERACTION_. MEMs were performed using Minitab version 20 and all graphs produced using GraphPad Prism software version 10.

Proteomic analysis results in large datasets requiring many statistical comparisons, increasing the risk of type I error. This is typically accounted for by Bonferroni correction, which works well for independent observations but is highly conservative. In the case of HDL proteomics, it was anticipated that identified proteins would likely be correlated with one another and therefore not independent, rendering Bonferroni correction a poor option in this instance. Data resulting from proteomic analysis of HDL fractions were therefore first analysed by orthogonal projections of latent structures-discriminant analysis (OPLS-DA), incorporating unit variance scaled demographic, anthropometric and proteomic data to investigate the differences between the groups under study. Variables with a variable influence on projection (VIP) value > 1.0 and VIP value > standard error were included in the analysis to find only those most important to group separation. Model quality was evaluated using *R*2 and *Q2,* describing the goodness of fit and prediction, respectively, and Analysis of Variance of Cross-Validated predictive residuals (CV-ANOVA). OPLS-DA was considered statistically significant at *P*<0.05 and was performed using SIMCA software version 18. OPLS-DA models were followed up with univariate analyses of each individual protein identified by nLC-MS/MS. This was performed using MEM, as data were non-parametric and the groups were unbalanced [20]. MEMs were deemed valid if there was a normal distribution of residuals. Where residuals were not normally distributed, and the input data contained a large proportion of zeros, such as with proteomic LFQ intensities, the data were imputed by adding the minimum non-zero value (an estimate of the limit of detection) to all data points before log-transforming in order to maintain the scale of the data [21].

## Results

### Metabolic and anthropometric changes with weight gain in white Europeans and South Asians

A summary of the ethnic differences in metabolic changes is presented here for context (all reported as mean ± standard error of the mean); full demographic and anthropometric data for the GlasVEGAS study have been previously published by McLaren et al. [14]. EUs (22 ± 0.7 years) and SAs (23 ± 0.9 years) were similarly aged and similarly increased their BMI with weight gain (+1.41 ± 0.06 kg/m^2^ and +1.41 ± 0.08 kg/m^2^, respectively). Fasting glucose and postprandial glucose were not different by ethnicity or with weight gain. Fasting insulin after weight gain was unchanged in the EUs (baseline 6.1 ± 1.1 µU/ml, +0.0 ± 1.0 µU/ml with weight gain) but increased in SAs (baseline 6.0 ± 0.9 µU/ml, +10.5 ± 5.1 µU/ml with weight gain, *P*=0.02). Postprandial insulin in the EUs was modestly increased by weight gain (baseline 28.2 ± 2.7 µU/ml, +4.4 ± 2.5 µU/ml with weight gain, *P*=0.01) but substantially increased with weight gain in SAs (baseline 45.6 ± 6.1 µU/ml, +17.8 ± 5.0 µU/ml with weight gain, *P*=0.01). Fasting and postprandial triglycerides were both higher in SAs compared with the EUs (fasting 1.09 ± 0.14 mmol/l versus 0.81 ± 0.07 mmol/l, respectively, *P*=0.05 and postprandial 1.52 ± 0.22 mmol/l versus 1.03 ± 0.09 mmol/l, respectively, *P*=0.02). Weight gain induced an increase in fasting and postprandial triglycerides in SAs and EUs overall (fasting +0.28 ± 0.13 mmol/l and +0.14 ± 0.07 mmol/l, respectively, *P*=0.0006 and postprandial +0.29 ± 0.15 mmol/l and +0.20 ± 0.09 mmol/l, respectively, *P*=0.0005). In terms of body composition, EUs had greater whole-body lean tissue at baseline than SAs (44.0 ± 1.2 l compared with 39.2 ± 1.2 l, *P*=0.01) and gained more lean tissue volume with weight gain (+2.4 ± 0.5 l and +0.7 ± 0.3 l, *P*=0.02). SAs had both greater abdominal subcutaneous adipose tissue (4.0 ± 0.4 l compared with 2.5 ± 0.2 l, *P*=0.0007) and liver fat fraction (4.1 ± 1.0% compared with 2.1 ± 0.3 %, *P*=0.03) at baseline than the EUs. Weight gain did not statistically increase liver fat fraction, though there was high inter-individual variability in the SAs (EU, +1.0 ± 0.9 %; SA, 3.6 ± 3.3 %). Finally, while VAT volume was similar at baseline in both ethnicities (EU, 1.1 ± 0.1 l; SA, 1.4 ± 0.2 l), EUs increased the volume of this depot to a greater degree with weight gain (EU, +0.6 ± 0.1 l; SA, 0.3 ± 0.1 l; *P*=0.004).

### HDL apoA-I, cholesterol content and subclass distribution in white Europeans and South Asians

**Figure 1:**
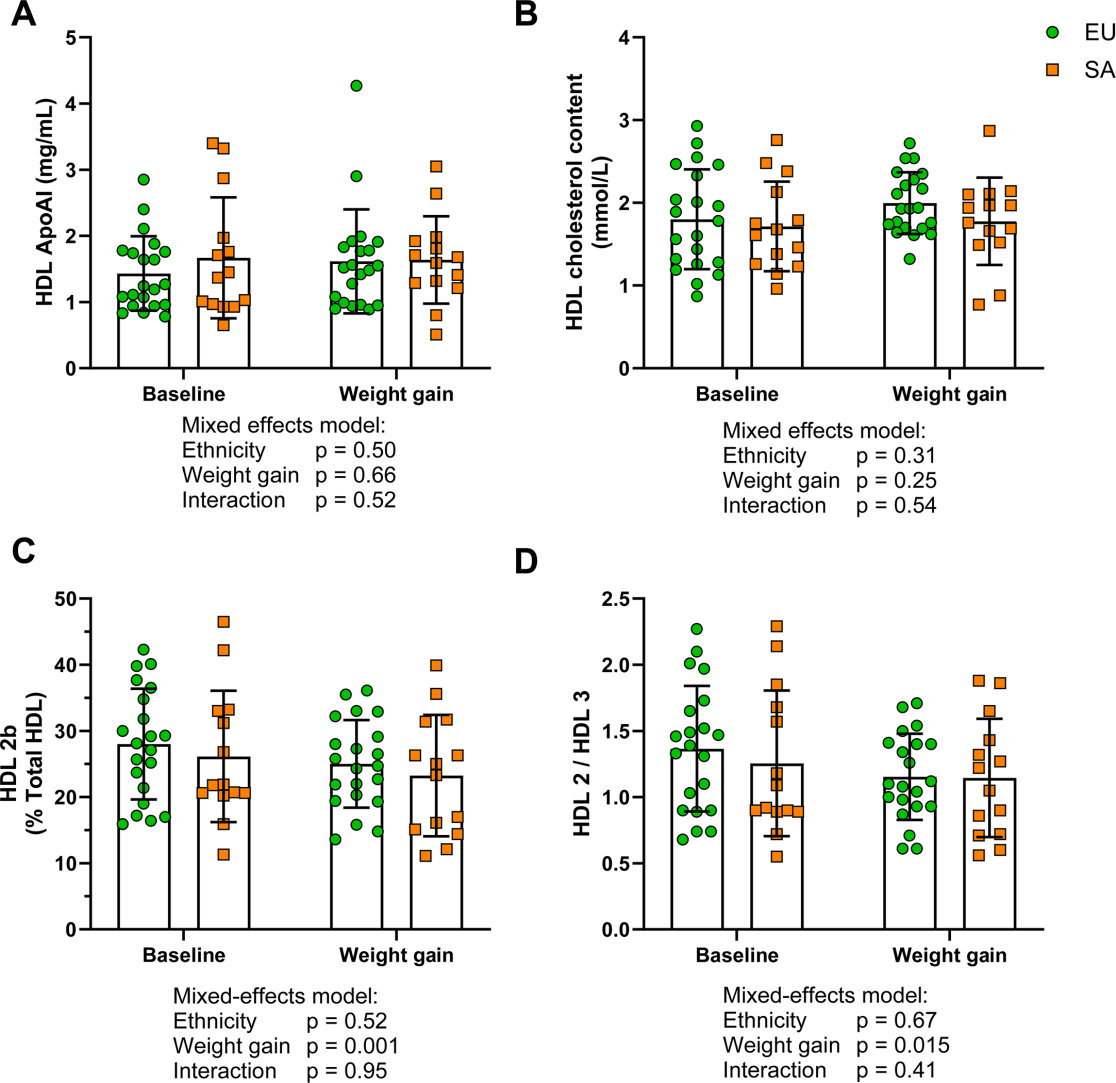
Measures of HDL constituents and subclass distribution in the GlasVEGAS study. **(A**) HDL apoA-I content, (**B**) HDL total cholesterol content, (**C**) proportion of HDL 2b and (**D**) ratio of HDL 2 to HDL 3 in white Europeans (EUs, *n*=21) and South Asians (SAs, *n*=14) before and after weight gain. Data expressed as mean ± standard deviation. Data were analysed by mixed effects model, and statistical significance was assumed at *P*<0.05. Apo, apolipoprotein; HDL, high-density lipoprotein.

EU and SA HDL responded similarly to weight gain, with unchanged HDL apoA-I and total cholesterol content (Figure 1A and B). The proportion of HDL 2b was not different between EUs and SAs but was reduced by weight gain (Figure 1C). The HDL 2/3 ratio, reflective of overall HDL size, was decreased by weight gain but not different between the two ethnicities (Figure 1D). Full details of HDL subclass distribution are in Supplementary Figures S1 and S2.

### HDL proteomic composition in white Europeans and South Asians before and after weight gain

**Figure 2:**
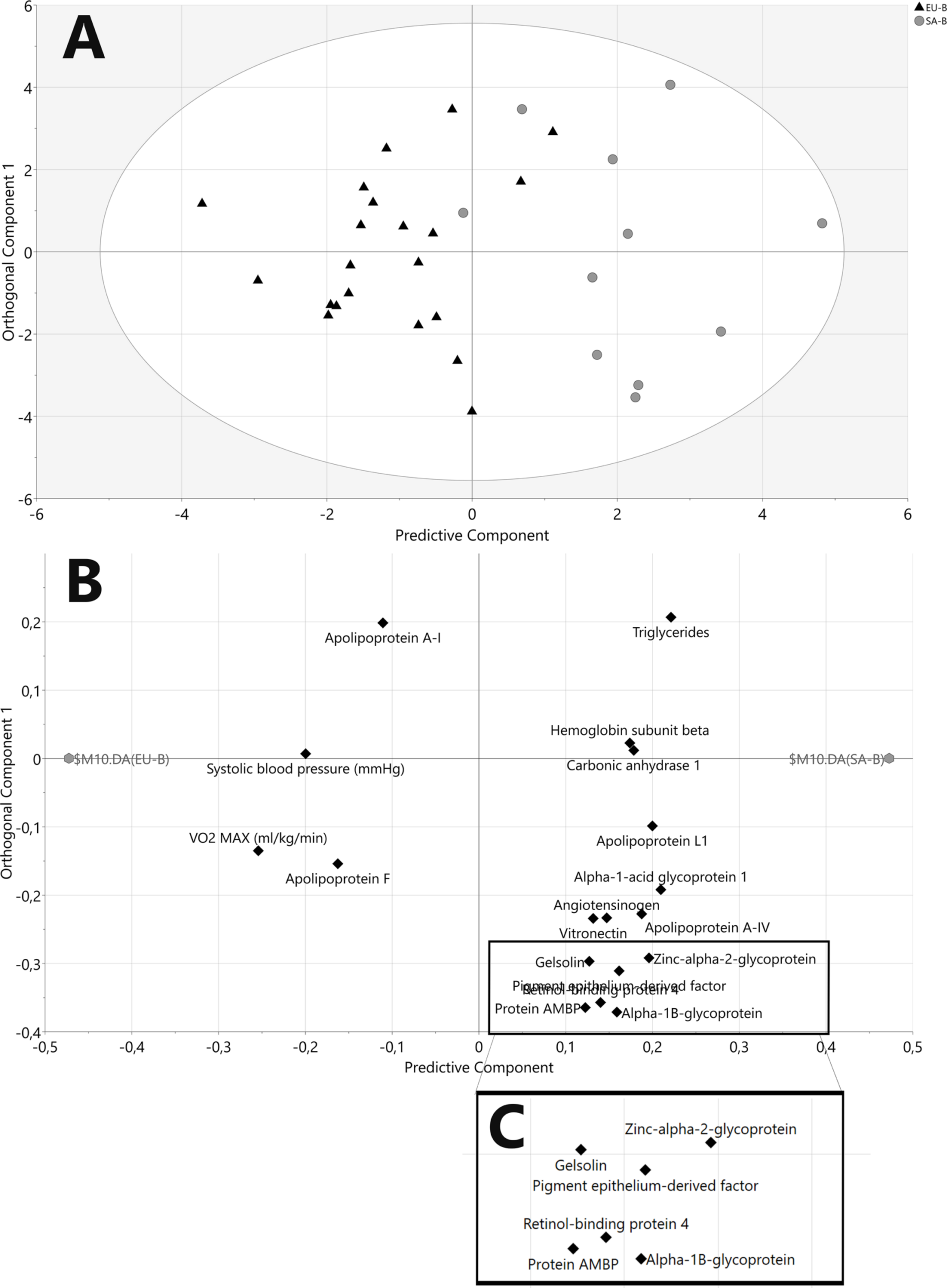
OPLS-DA model of white Europeans and South Asians at baseline, including demographics and identified HDL proteins. **(A**) Scores plot of OPLS-DA. The x-axis represents between-group variation, and the y-axis represents within-group variation. *R2X=0.46, R2Y=0.65, Q2=0.45, CV-ANOVA P=0.002*. The ellipse represents the confidence region of a Hotelling’s T2 test with a significance level of *P*=0.05. Samples outside the ellipse may be considered as potential outliers. Black triangles=EU, grey circles=SA. (**B**) OPLS-DA loadings plot. This plot shows the correlations of variables with one another and their impact on the separation of the groups. Variables located close together are correlated. Variables closer to the group identifiers are more important to the observed separation between EU (left) and SA (right). (**C**) Crop of B for clarity. EU, *n*=21, SA, *n*=11.

**Figure 3:**
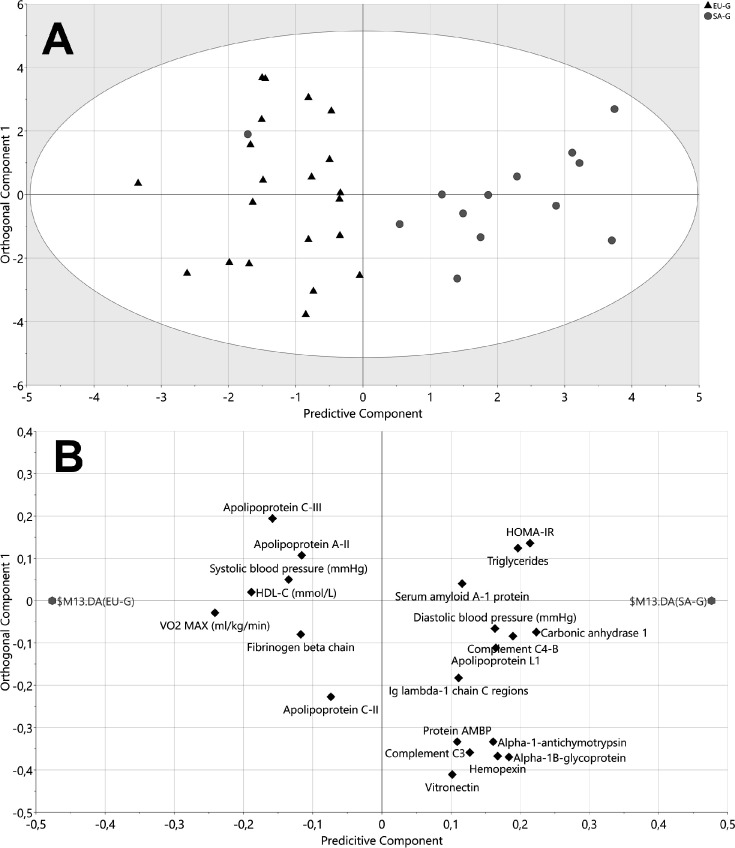
OPLS-DA model of white Europeans and South Asians after weight gain including demographics and identified HDL proteins. **(A**) Scores plot of OPLS-DA. The x-axis represents between-group variation, and the y-axis represents within-group variation. *R2X=0.36, R2Y=0.66, Q2=0.41, CV-ANOVA P=0.003.* The ellipse represents the confidence region of a Hotelling’s T2 test with a significance level of *P*=0.05. Samples outside the ellipse may be considered as potential outliers. Black triangles=EU, grey circles=SA. (**B**) OPLS-DA loadings plot. This plot shows the correlations of variables with one another and their impact on the separation of the groups. Variables located close together are correlated. Variables closer to the group identifiers are more important to the observed separation between EU (left) and SA (right). EU, *n*=21. SA, *n*=13.

Proteomic analysis of HDL revealed 149 proteins in at least one HDL sample (listed in Supplementary Table S1). Of these, 50 proteins were present in >50% of HDL samples and were deemed HDL-associated (full details in Supplementary Table S2. An OPLS-DA model containing all demographics and HDL-associated proteins showed clear separation between EUs (to the left of the scores plot) and SAs at baseline (to the right of the scores plot) (Figure 2A). The loadings plot (Figure 2B) indicated that three demographic factors, in addition to plasma triglycerides and a constellation of HDL proteins, were most different between the ethnicities at baseline. ApoA-I and apoF were higher in EUs, while apoA-IV, zinc-alpha-2-glycoprotein (ZAG) and apoL-1 were higher in SAs. A further OPLS-DA model comparing EUs and SAs after weight gain also showed clear separation between the two groups (Figure 3A). The corresponding loadings plot indicated that VO_2_ max, HDL-C and apoA-II and apoC-III were higher in EUs after weight gain, while HOMA_IR_, SAA-1 and immune-related proteins such as complement C4-B were higher in SAs after weight gain (Figure 3B).

**Figure 4:**
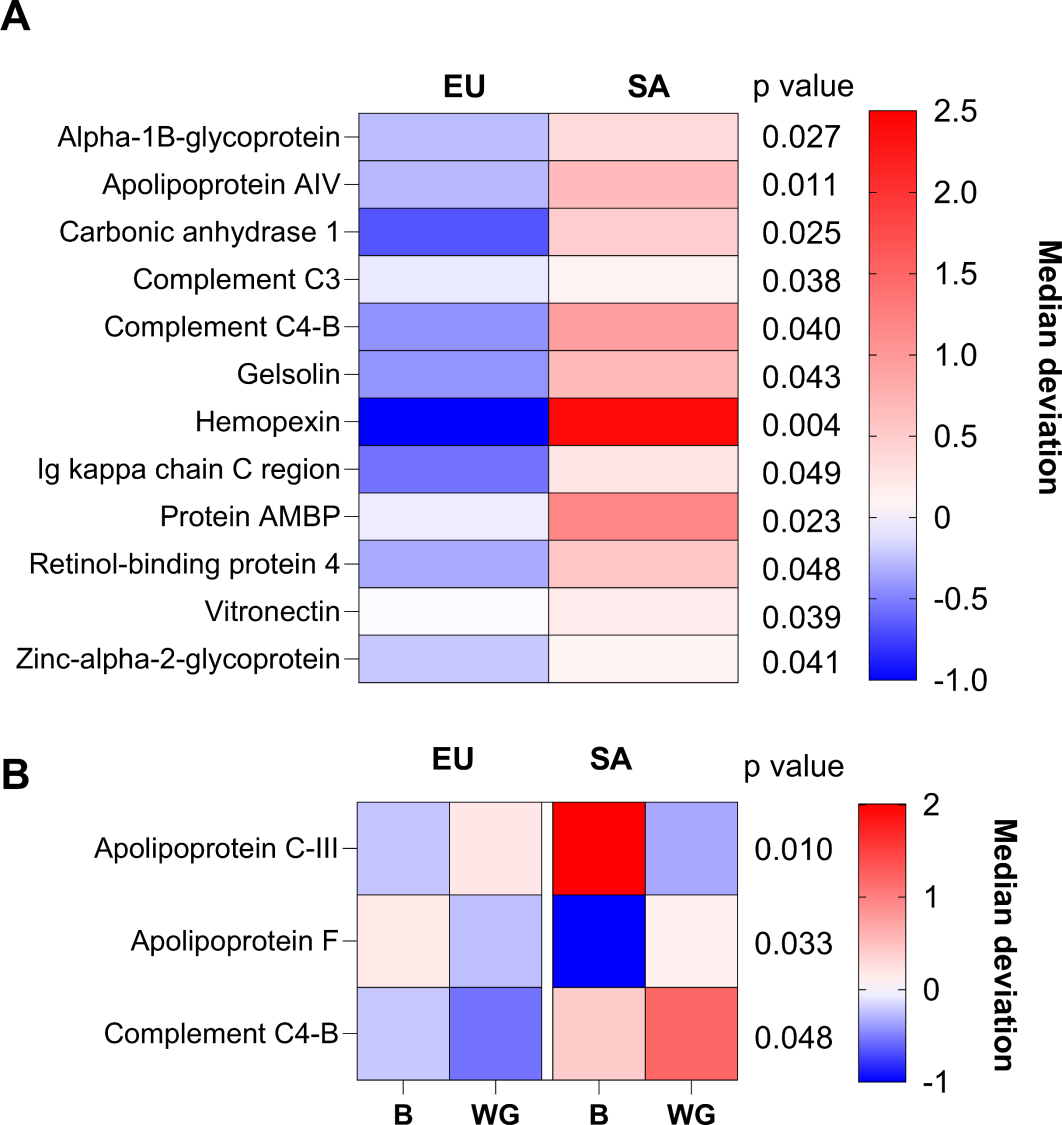
Heatmap of proteins identified on HDL with significant differences between white Europeans and South Asians before and after weight gain. **(A**) Heatmap of proteins with significant differences by ethnicity. (**B**) Heatmap of proteins with significant interactive (ethnicity × weight gain) differences between Europeans and South Asians. Heatmaps generated with median absolute deviation standardised LFQ intensities. *n*=21 EUs at baseline and weight gain, *n*=11 SAs at baseline and *n*=13 with weight gain. EU, European; SA, South Asian; B, baseline; WG, weight gain.

**Figure 5:**
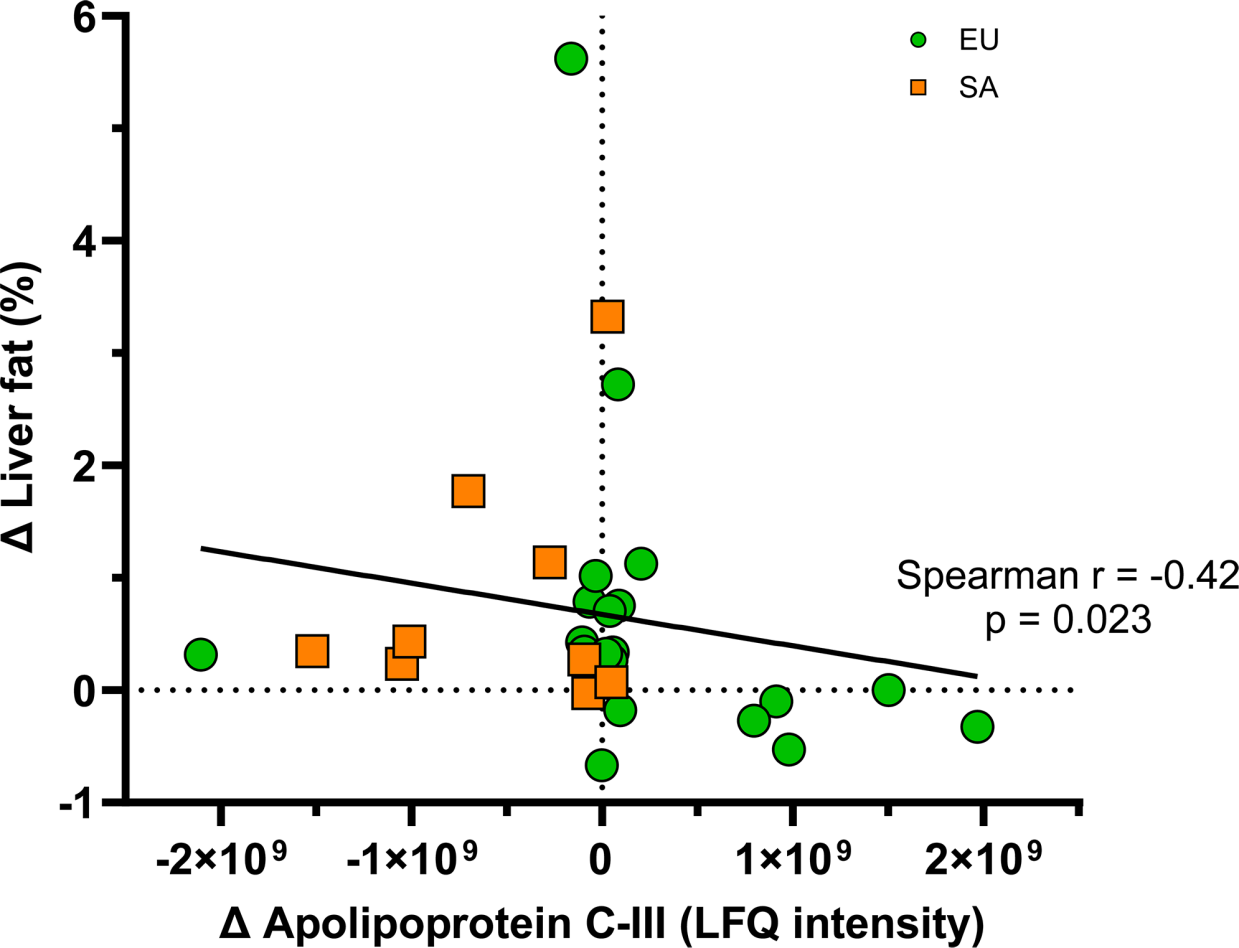
Correlation between change in HDL apoC-III and change in liver fat fraction after weight gain. Correlation was assessed by Spearman’s rank test. Statistical significance was assumed at *P*<0.05. EU, European, green circles, *n*=21; SA, South Asian, orange squares, *n*=11; LFQ, label-free quantification.

Of the 50 identified HDL proteins, 15 had significant differences when comparing the EU and SA response to weight gain in follow-up MEMs. These included apolipoproteins, immune-related proteins and adipokines. Individual plots of each protein can be found in Supplementary Figures S3 and S4. There were four apolipoproteins with significant differences in their LFQ intensities on HDL when comparing EUs and SAs. ApoA-IV LFQ intensity was higher on SA HDL (Figure 4A). There was an ethnicity × weight gain interaction in apoC-III and apoF (Figure 4B). The LFQ intensity of HDL apoC-III increased in EUs but decreased in SAs after weight gain, while the LFQ intensity of HDL apoF decreased in EUs but increased in SAs post weight gain. As these apolipoproteins are involved in triglyceride handling, the relationship between the change in these proteins after weight gain and the change in liver fat fraction was explored. Only the change in apoC-III with weight gain correlated with the change in liver fat with weight gain (Spearman *r*=-0.42, *P*=0.023, Figure 5). Five proteins identified on HDL with differences when comparing EUs and SAs were immune-related. The LFQ intensities of complement C3, gelsolin, hemopexin and Ig kappa chain c region were all higher in SA HDL compared with EU HDL irrespective of weight gain (Figure 4A). The LFQ intensity of complement C4-B was also higher in SA HDL compared with EU HDL. After weight gain, complement C4-B was significantly higher in SA HDL compared with EU HDL. Two adipokines identified on HDL when comparing EUs and SAs had significant differences. The LFQ intensity of both retinol-binding protein 4 (RBP4) and ZAG was higher in SA HDL compared with EU HDL irrespective of weight gain (Figure 4A). Finally, four further miscellaneous proteins were significantly higher in the HDL of SAs compared with EUs irrespective of weight gain: alpha-1B-glycoprotein, carbonic anhydrase 1, protein AMBP and vitronectin (Figure 4A).

### HDL S1P content, but not its carrier apoM, was lower in South Asian HDL compared with white Europeans

**Figure 6:**
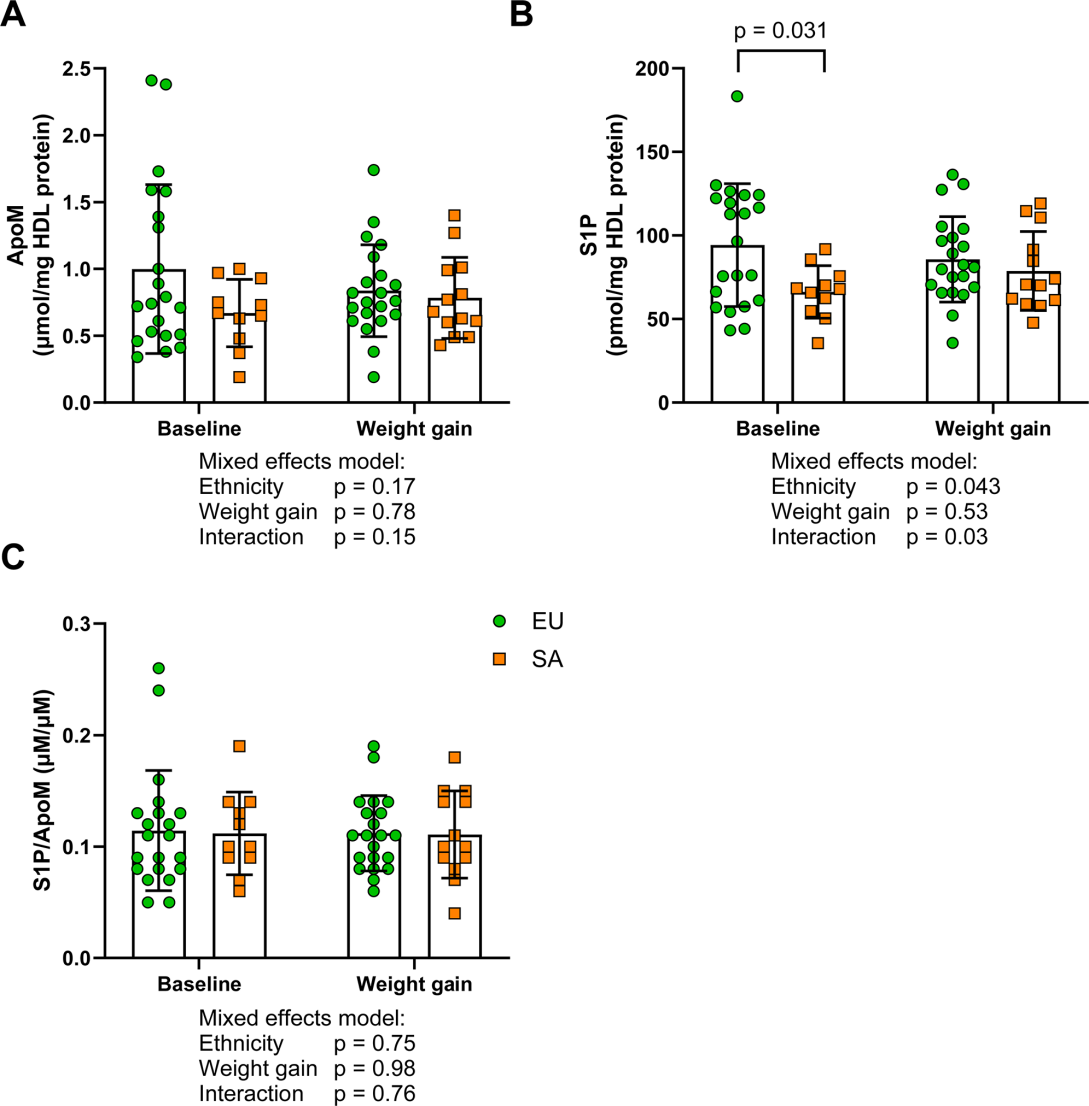
HDL apoM and S1P content in white Europeans and South Asians before and after weight gain. **(A**) ApoM content of HDL per mg of HDL protein, (**B**) S1P content of HDL per mg of HDL protein and (**C**) the ratio of S1P/apoM of HDL in Europeans (EUs, *n*=21) and South Asians (SAs, *n*=14) before and after weight gain. Data expressed as mean ± standard deviation. Data were analysed by mixed effects models, and statistical significance was assumed at *P*<0.05. ApoM, apolipoprotein M; S1P, sphingosine-1-phosphate; HDL, high-density lipoprotein.

HDL apoM content was numerically lower in SA HDL overall and was unaffected by weight gain in either ethnicity (Figure 6A). However, S1P content was lower in SAs compared with EU HDL overall (73.0 ± 20.9 compared with 90.0 ± 31.6 pmol/mg HDL protein, *P*=0.043, mean ± standard deviation), and HDL S1P was significantly lower in SAs at baseline (66.2 ± 15.7 compared with 94.3 ± 31.6 pmol/mg HDL protein, *P*=0.031, Figure 6B). The ratio of S1P to apoM was not different by ethnicity or with weight gain (Figure 6C).

### *In vitro* paraoxonase-1 activity and anti-inflammatory measures of HDL were not different between white Europeans and South Asians irrespective of weight gain

**Figure 7:**
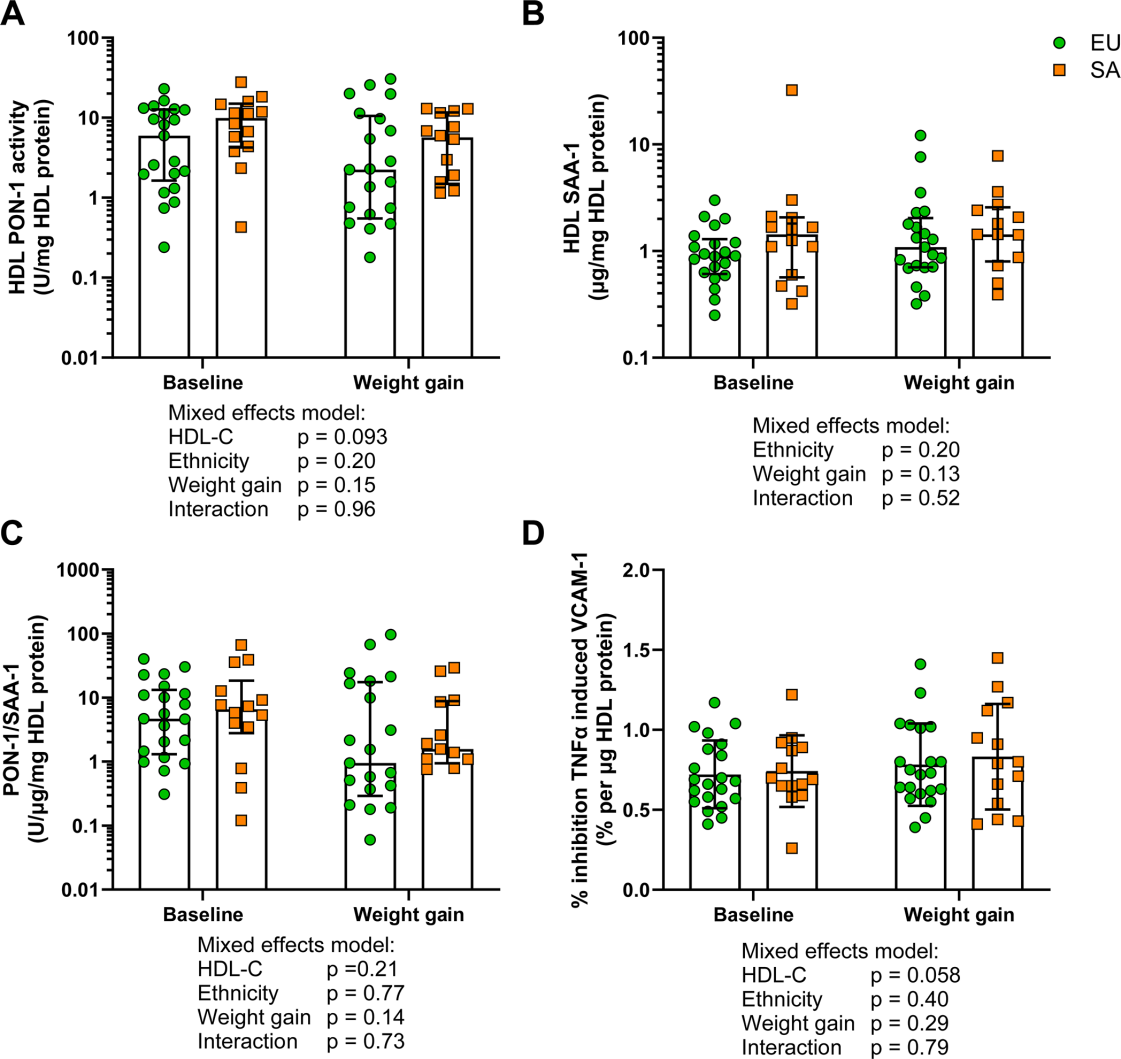
HDL paraoxonase-1 and anti-inflammatory *in vitro* function in white Europeans and South Asians before and after weight gain. **(A**) Paraoxonase-1 (PON-1) activity of HDL, (**B**) serum amyloid A1 (SAA-1) content of HDL and (**C**) the ratio of PON-1 to SAA-1 on HDL in Europeans (EUs, *n*=21) and South Asians (SAs, *n*=14) before and after weight gain. Data expressed as median ± interquartile range and shown on a log10 axis for clear visualisation. (**D**) % inhibition of TNFα-induced VCAM-1 expression by HDL in endothelial cells in EUs (*n*=21) and SAs (*n*=14) before and after weight gain. Data expressed as mean ± standard deviation. Comparisons were made in A-D by the mixed effects model. Statistical significance was assumed at *P*<0.05.

HDL PON-1 activity was not different by ethnicity or after weight gain (Figure 7A). SAA-1 content of HDL was numerically higher in SAs but not statistically different compared with EUs (Figure 7B). The ratio of PON-1 activity to SAA-1 content in HDL from EUs and SAs did not differ by ethnicity or weight gain (Figure 7C). The anti-inflammatory function of HDL was not different by ethnicity or weight gain (Figure 7D).

## Discussion

This study set out to understand the differences in HDL composition and function in young and healthy EU and SA men, at baseline and in response to weight gain, in a longitudinal study design. SA HDL apoA-I, total cholesterol, total protein and subclass distribution were all unchanged compared with EUs. This is consistent with the findings of Bakker et al. [12], who found no differences in HDL composition between EUs and SAs, both young and middle-aged, by nuclear magnetic resonance (NMR) spectrometry. In contrast, the present study found marked differences in HDL protein composition between EUs and SAs, both at baseline and in response to weight gain, particularly with respect to lipid metabolism-related proteins. However, these differences did not influence the HDL *in vitro* functions investigated in this study. This suggests that HDL protein composition is a putative marker of impaired lipid metabolism in SAs, which was evident despite the modest degree of weight gain and the young age of the participants.

OPLS-DA models comparing EUs and SAs at baseline and after weight gain showed clear separation between the ethnicities and suggested that HDL proteins contributed significantly to the observed separation alongside demographic measures. Four apolipoproteins were significantly different between EUs and SAs. SAs had higher HDL apoA-IV, which might suggest an altered response to dietary fat intake or an ethnic difference in the contribution of intestinally derived HDL to the overall HDL pool [22]. ApoA-IV production is increased in the intestine in response to the ingestion of fat [23], which implies either that SAs are hyper-responsive in this regard or consume a fat-rich diet, though this data was not collected. ApoA-IV transfers to HDL through interaction with chylomicron remnants, so increased apoA-IV on HDL could indicate increased chylomicron lipolysis. ApoA-IV increases the activity of cholesteryl ester transfer protein (CETP) similarly to apoA-I [24], which suggests increased remodelling and turnover of HDL in SAs. There is evidence that apoA-IV increases insulin secretion by pancreatic β-cells in response to glucose [25]. SAs in this cohort were insulin resistant, as seen in the elevated fasting and postprandial insulin concentrations after weight gain; the increase in HDL apoA-IV may be a compensatory mechanism to maintain glucose homeostasis.

**Figure 8:**
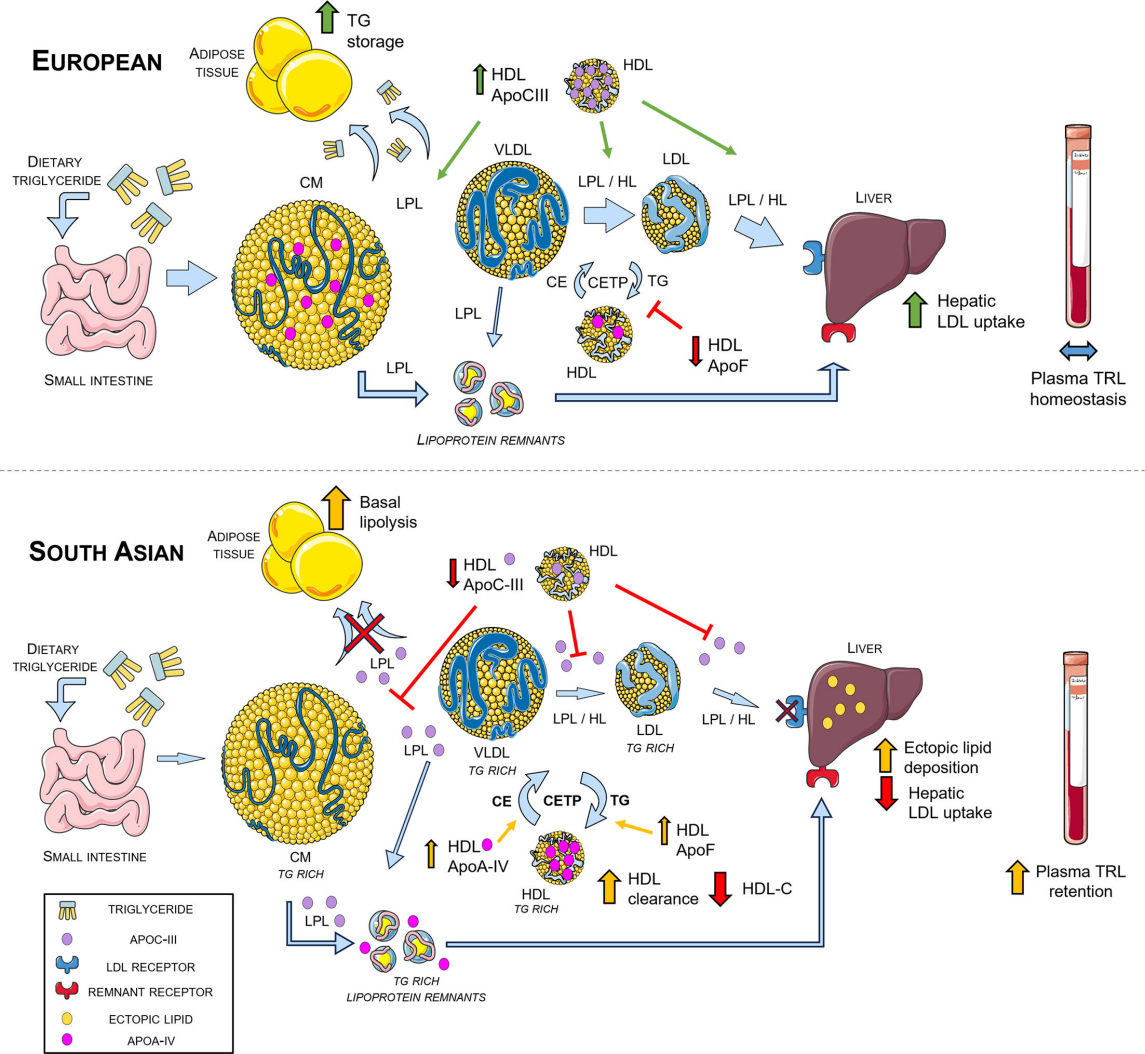
Proposed model of differences in lipid metabolism after weight gain in white European and South Asian men. Top: After weight gain in Europeans, increased HDL apoC-III leads to increased lipase activity, while decreased HDL apoF and apoA-IV result in the inhibition of CETP. These mechanisms work together to maintain plasma lipoprotein homeostasis. Bottom: In South Asians, weight gain decreases HDL apoC-III, resulting in reduced lipase activity. Reduced lipase activity results in the formation of triglyceride-rich remnants, which are taken up by the liver. Increased CETP activity due to increased HDL apoA-IV and apoF results in triglyceride-enriched HDL and reduced hepatic LDL uptake. Taken together, these changes result in increased plasma triglyceride-rich lipoprotein retention, which when coupled with increased basal adipocyte lipolysis, results in hepatic fat deposition. ApoA-IV, apolipoprotein AIV; ApoC-III, apolipoprotein CIII; ApoF, apolipoprotein F; CM, chylomicron; LPL, lipoprotein lipase; VLDL, very low-density lipoprotein; HL, hepatic lipase; CE, cholesteryl ester; CETP, cholesteryl ester transfer protein; HDL, high-density lipoprotein; LDL, low-density lipoprotein; TG, triglyceride; TRL, triglyceride-rich lipoprotein.

Weight gain increased the lipoprotein lipase (LPL) inhibitor apoC-III in EU HDL but reduced it in SA HDL. ApoC-III is active in inhibiting lipoprotein and hepatic lipase when on triglyceride-rich lipoproteins (TRLs) like chylomicrons, VLDL and LDL, with HDL acting as a reservoir and distributor of apoC-III among lipoproteins (reviewed in [26]). This finding suggests that with weight gain in EUs, apoC-III is sequestered on HDL to increase the activity of LPL and hepatic lipase, therefore maintaining plasma triglyceride processing in response to increased dietary lipid. In SAs, this fails to happen; weight gain decreased HDL apoC-III, suggesting lipase activity is decreased, therefore increasing retention of TRLs in plasma. The change in apoC-III with weight gain negatively correlated with the change in liver fat fraction, which implies that excess triglyceride in SAs is deposited in the liver as a compensatory mechanism, despite the young age and good health of these participants. Finally, HDL apoF tended to decrease in EU HDL but increase in SA HDL with weight gain. HDL-bound apoF activates CETP; a decrease in HDL apoF suggests CETP inhibition and increased hepatic LDL uptake to counteract rising plasma triglycerides. It appears that this protective mechanism does not occur in SAs, and our finding of increased HDL apoF corroborates reports of higher HDL CETP activity in this ethnicity [27]. HDL lipid composition was not performed in this study, but increased CETP activity in SAs may enrich HDL with triglyceride as a pathogenic compensatory response to rising plasma triglycerides. Taken together, these changes in HDL apolipoproteins and remodelling proteins may reflect altered lipoprotein metabolism in SAs that contributes to increased CETP activity and the observation that at a lower BMI, SAs have increased ectopic liver fat compared with EUs [7]. This altered lipoprotein metabolism may contribute to the overall predisposition of SAs to develop T2DM, particularly as it has been observed in young and healthy men with a mean 1.5 kg/m^2^ increase in BMI. Our hypothesis for describing differences in lipid metabolism between EU and SA men with weight gain, based on these HDL proteomics data, is shown in Figure 8. This hypothesis requires further testing, using a combination of proteomic and lipidomic analysis of all lipoprotein subclasses, as well as measures of lipolytic enzyme activity in a similar study design.

The five immune-related proteins identified on HDL were all higher in SAs compared with EUs, irrespective of weight gain, suggesting that SAs have an increased immune response. Non-diabetic, healthy adult SAs have increased plasma levels of complement C3 [28] and C-reactive protein [29] compared with EU counterparts. As these two acute-phase reactants are increased in SAs, it can be inferred that the increase in inflammatory proteins on HDL is a function of the systemic increase in the plasma concentration of these proteins. The adipokines RBP4 and ZAG were both higher in SA HDL. RBP4 is increased with insulin resistance [30]; it increases adipocyte basal lipolysis and reduces the ability of insulin to suppress lipolysis (which also leads to ectopic liver fat accumulation) and has been implicated in immune stimulation [31]. The higher RBP4 in SA HDL corroborates SA predisposition to insulin resistance and may contribute to this phenotype [3]. The inflammatory nature of RBP4 may also partly explain the increased immune-related proteins on HDL; RBP4-treated macrophages secrete more TNFα, which in turn increases acute phase protein synthesis [31]. The higher ZAG on SA HDL may be a compensatory response to counteract the negative effects of RBP4 and adverse lipoprotein metabolism indicated by the altered apolipoprotein make-up of SA HDL.

S1P, carried on HDL bound to apoM, is well established as protective against CVD largely due to its vasoactive and cyto-protective endothelial signalling [32]. Our findings are similar to a previous study of the metabolic response to cold exposure in Dutch SA and EU men, where no ethnicity differences in plasma S1P or apoM were observed [33]. There is emerging preclinical evidence that S1P may also have a role in adipocyte function (including preadipocyte proliferation and maturation) and hepatic insulin sensitivity (reviewed in [34]). In the same cohort as the present study, McLaren et al. [14] reported differences between EUs and SAs in adipocyte size distribution and gene expression, notably larger adipocytes at baseline and a smaller decrease in the proportion of small adipocytes with weight gain in SAs, with lower expression of lipid metabolism genes and higher expression of adipocyte differentiation inhibitor genes. The link between the observed lower HDL S1P and unfavourable measures of adipocyte status in young and healthy SAs warrants further investigation. S1P can also be bound to albumin, where its bioactivity in the vasculature is reduced compared with HDL-bound S1P [35]. Lower HDL S1P may reflect a shift to albumin or to triglyceride-rich lipoproteins in SAs [36], though this was not measured in this study.

Whether corrected for HDL protein or HDL cholesterol content, PON-1 arylesterase activity did not differ by ethnicity or by weight gain. This finding matches the observed lack of difference in EU and SA serum PON-1 mass and activity in a study of middle-aged renal transplant patients [37]. Bakker et al. [12] studied both adolescent (similarly aged to the present cohort) and adult EU and SA men; a reduction in the antioxidant capacity of HDL was only observed in the older group of SA men based on HDL inhibition of LDL oxidation. It may be that moderate weight gain in young and otherwise healthy SA men is insufficient to provoke the impairment in the antioxidant capacity of HDL seen in older SA men. Similarly, Bakker et al. failed to observe a difference in HDL *in vitro* anti-inflammatory function between EU and SA adults and adolescents. The lack of difference in anti-inflammatory function seen in the present study was despite differences in HDL protein composition between the ethnicities, particularly the increased presence of immune and inflammatory proteins on SA HDL. A previous cross-sectional study performed in this laboratory failed to detect changes in HDL *in vitro* functions between control men and endurance athletes despite a favourable HDL protein composition in the latter, though changes in function were observed between these groups and men with impaired glucose regulation [10]. There we argued that HDL is a scavenger particle with a changing composition reflective of the current physiology, and with a functional ceiling in healthy individuals. This study lends further weight to this argument given the healthy status of the participants even with moderate weight gain.

The primary strength of this study was the repeated measures design, allowing each individual to be their own control, adding statistical power to detect changes in the variables measured. The acute effects of altered diet or exercise on HDL were accounted for by an energy-neutral diet for three days prior to each visit. Much of the research into HDL composition and function with insulin resistance focuses on cross-sectional studies comparing controls and overweight/obese individuals. This study aimed to address this gap in the literature by focusing on weight gain-induced changes in HDL composition and function in otherwise young and healthy EU and SA men. However, the GlasVEGAS study was powered based on a one standard deviation difference between the groups. This is a large difference, which may have masked subtle HDL differences due to the study population being too small, such as our findings on S1P and apoM, and that SAA-1 contributed to separation by ethnicity in the OPLS-DA model but was not significantly different when assessed by univariate statistics. A previous study measuring HDL proteomic composition in endotoxemia found statistically significant differences with *n*=10 per group [38]. Many published studies describe overweight or obese study groups, while even after weight gain the participants in the GlasVEGAS study were still of a healthy weight. It may be that the weight gain was insufficient to reveal changes in HDL function or subclass distribution, though changes in HDL protein composition were readily detected. It would be unethical to induce more serious weight gain in young healthy men, especially in the SA cohort, who are particularly susceptible to weight gain-induced complications. There is some uncertainty around the agreement between lipoprotein subclass measured by gel electrophoresis and other methods such as NMR-based lipidomics, with no clear consensus in the literature [39–41]. It is likely, however, that the more detailed NMR-based lipidomic analyses can detect subtle differences in particle size. Due to the large number of measures and biopsies taken in the original study, it was not feasible to include *in vivo* measures of vascular function such as flow-mediated dilation. The cholesterol efflux capacity of HDL and measurement of the other plasma lipoproteins was not performed in this study. It was therefore not possible to investigate the role of the lipoprotein metabolism-associated proteins on HDL in its efflux capacity and the consequences for other lipoproteins and vascular function. Finally, this study was performed in young, healthy weight men to investigate early metabolic responses to weight gain. As such, these findings should not be generalised to women or older populations or individuals with obesity. The original study by McLaren et al. did not recruit female participants due to the added complexity of controlling for the menstrual cycle (which alters metabolic response) and the need for a greater degree of weight gain to induce metabolic disturbance [14]. Future studies including female EUs and SAs may reveal both sex and ethnicity dependent differences in the HDL response to weight gain.

In conclusion, this study revealed that at baseline and with moderate weight gain, HDL protein composition differed between EU and SA HDL without changes detected in HDL function, suggesting that HDL acts as a marker of aberrant lipoprotein metabolism and triglyceride handling in SAs. This may offer a potential mechanism explaining the increased ectopic liver fat observed in this ethnicity, warranting further investigation. Future studies might focus on specific interactions between the different lipoproteins and the liver in a similar cohort, such as using stable isotope-labelled fatty acids (in a similar manner to [42]). These changes occurred in young, healthy men with an average 1.5-unit increase in BMI, providing some context to the vastly different age and BMI of onset of T2DM and the increased cardiometabolic risk in SAs. In its scavenger role, HDL may be a passive carrier of a number of proteins that reflect systemic physiology and wider metabolism, rather than HDL protein composition being directly linked to its vascular functions.

## Supplementary material

online supplementary material 1.

## Data Availability

Anonymised data generated in the present study will be available on request. The original complete dataset contains a number of indirect identifiers and therefore cannot be shared publicly in line with current best practice [43].

## Abbreviations

Apo: apolipoprotein
BMI: body mass index
CETP: cholesteryl ester transfer protein
CVD: cardiovascular disease
ELISA: enzyme-linked immunosorbent assay
EU: European
GlasVEGAs: Glasgow visceral & ectopic fat with weight gain in South Asians
HDL: high-density lipoprotein
HDL-C: high-density lipoprotein-cholesterol
LC-MS/MS: liquid chromatography tandem mass spectrometry
LDL: low-density lipoprotein
LFQ: label-free quantification
LPL: lipoprotein lipase
MEM: mixed effects model
NMR: nuclear magnetic resonance
OPLS-DA: orthogonal projections of latent structures-discriminant analysis
PON-1: paraoxonase-1
RBP4: retinol-binding protein 4
RF: retention factor
SA: South Asian
SAA-1: serum amyloid A1
S1P: sphingosine-1-phosphate
T2DM: type 2 diabetes mellitus
TNFα: tumour necrosis factor alpha
TRL: triglyceride-rich lipoprotein
VAT: visceral adipose tissue
VCAM-1: vascular cell adhesion molecule-1
VIP: variable influence on projection
VLDL: very-low density lipoprotein
ZAG: zinc-alpha-2-glycoprotein
nLC-MS/MS: nano liquid-chromatography tandem mass spectrometry
