# Supplementary material for: HDL protein composition differs between young white European and South Asian men before and after weight gain
Source: Clin Sci (Lond). 2025 Dec 18;139(24):1659–74. doi: 10.1042/CS20258040 (PMC12794315; doi:10.1042/CS20258040)
Supplement: online supplementary material 1. [file cs-139-24-CS20258040-s001.pdf]

Supplemental Data Table 1: Proteins identified in at least one HDL sample

Gene names	Protein IDs	Protein names
A1BG	P04217	Alpha-1B-glycoprotein
ACTBL2	Q562R1	Beta-actin-like protein 2
ACTG1	P63261	Actin, cytoplasmic 2
ACTN1	P12814	Alpha-actinin-1
AGT	P01019	Angiotensinogen
AHSG	P02765	Alpha-2-HS-glycoprotein
ALB	P02768	Serum albumin
ALDOA	P04075	Fructose-bisphosphate aldolase A
ALOX12	P18054	Arachidonate 12-lipoxygenase, 12S-type
AMBP	P02760	Protein AMBP
ANTXR2	P58335	Anthrax toxin receptor 2
APMAP	Q9HDC9	Adipocyte plasma membrane-associated protein
APOA1	P02647	Apolipoprotein A-I
APOA2	P02652	Apolipoprotein A-II
APOA4	P06727	Apolipoprotein A-IV
APOA5	Q6Q788	Apolipoprotein A-V
APOB	P04114	Apolipoprotein B-100
APOC1	P02654	Apolipoprotein C-I
APOC2	P02655	Apolipoprotein C-II
APOC3	P02656	Apolipoprotein C-III
APOC4	P55056	Apolipoprotein C-IV
APOD	P05090	Apolipoprotein D
APOE	P02649	Apolipoprotein E
APOF	Q13790	Apolipoprotein F
APOH	P02749	Beta-2-glycoprotein 1
APOL1	O14791	Apolipoprotein L1
APOM	O95445	Apolipoprotein M
ARG1	P05089	Arginase-1
AZGP1	P25311	Zinc-alpha-2-glycoprotein
B2M	P61769	Beta-2-microglobulin
BLMH	Q13867	Bleomycin hydrolase
BPIFB1	Q8TDL5	BPI fold-containing family B member 1
C1S	P09871	Complement C1s subcomponent
C3	P01024	Complement C3
C4A	P0C0L4	Complement C4-A
C4B	P0C0L5	Complement C4-B
C9	P02748	Complement component C9
CA1	P00915	Carbonic anhydrase 1
CA2	P00918	Carbonic anhydrase 2
CA6	P23280	Carbonic anhydrase 6
CALM3	P0DP25	Calmodulin-3
CAMP	P49913	Cathelicidin antimicrobial peptide
CAP1	Q01518	Adenylyl cyclase-associated protein 1
CETP	P11597	Cholesteryl ester transfer protein
CFB	P00751	Complement factor B
CFD	P00746	Complement factor D
CFL1	P23528	Cofilin-1
CLEC3B	P05452	Tetranectin
CLIC1	O00299	Chloride intracellular channel protein 1
CLU	P10909	Clusterin
CNDP1	Q96KN2	Beta-Ala-His dipeptidase
DSG1	Q02413	Desmoglein-1
F2	P00734	Prothrombin
FERMT3	Q86UX7	Fermitin family homolog 3
FGA	P02671	Fibrinogen alpha chain
FGB	P02675	Fibrinogen beta chain

cont.

Gene names	Protein IDs	Protein names
FLG2	Q5D862	Filaggrin-2
FLNA	P21333	Filamin-A
GAPDH	P04406	Glyceraldehyde-3-phosphate dehydrogenase
GC	P02774	Vitamin D-binding protein
GPLD1	P80108	Phosphatidylinositol-glycan-specific phospholipase D
GSN	P06396	Gelsolin
GYPA	P02724	Glycophorin-A
HAL	P42357	Histidine ammonia-lyase
HBA1	P69905	Hemoglobin subunit alpha
HBB	P68871	Hemoglobin subunit beta
HBD	P02042	Hemoglobin subunit delta
HBG2	P69892	Hemoglobin subunit gamma-2
HLA-A	P04439	HLA class I histocompatibility antigen, A-3 alpha chain
HLA-C	P10321	HLA class I histocompatibility antigen, Cw-7 alpha
HP	P00738	Haptoglobin
HPR	P00739	Haptoglobin-related protein
HPX	P02790	Hemopexin
HRG	P04196	Histidine-rich glycoprotein
HSPA7	P48741	Putative heat shock 70 kDa protein 7
IGFALS	P35858	Insulin-like growth factor-binding protein complex acid
IGHA1	P01876	Ig alpha-1 chain C region
IGHG1	P01857	Ig gamma-1 chain C region
IGHG2	P01859	Ig gamma-2 chain C region
IGHG3	P01860	Ig gamma-3 chain C region
IGHG4	P01861	Ig gamma-4 chain C region
IGHM	P01871	Ig mu chain C region
IGKC	P01834	Ig kappa chain C region
IGLC1	P0DOY3	Ig lambda-1 chain C regions
IHH	Q14623	Indian hedgehog protein
ITGA2B	P08514	Integrin alpha-IIb
ITGB3	P05106	Integrin beta-3
ITIH2	P19823	Inter-alpha-trypsin inhibitor heavy chain H2
ITIH4	Q14624	Inter-alpha-trypsin inhibitor heavy chain H4
KNG1	P01042	Kininogen-1
LBP	P18428	Lipopolysaccharide-binding protein
LCAT	P04180	Phosphatidylcholine-sterol acyltransferase
LGALSL	Q3ZCW2	Galectin-related protein
LPA	P08519	Apolipoprotein(a)
LRG1	P02750	Leucine-rich alpha-2-glycoprotein
LUM	P51884	Lumican
MENT	Q9BUN1	Protein MENT
MMRN2	Q9H8L6	Multimerin-2
MYH9	P35579	Myosin-9
MYL6	P60660	Myosin light polypeptide 6
NSUN2	Q08J23	tRNA (cytosine(34)-C(5))-methyltransferase
ORM1	P02763	Alpha-1-acid glycoprotein 1
ORM2	P19652	Alpha-1-acid glycoprotein 2
PCYOX1	Q9UHG3	Prenylcysteine oxidase 1
PDLIM1	O00151	PDZ and LIM domain protein 1
PEX5L	Q8IYB4	PEX5-related protein
PF4	P02776	Platelet factor 4
PFN1	P07737	Profilin-1
PGLYRP2	Q96PD5	N-acetylmuramoyl-L-alanine amidase
PLTP	P55058	Phospholipid transfer protein
PON1	P27169	Serum paraoxonase/arylesterase 1
PON3	Q15166	Serum paraoxonase/lactonase 3
PPBP	P02775	Platelet basic protein
PRDX2	P32119	Peroxiredoxin-2

cont.

Gene names	Protein IDs	Protein names
PRDX6	P30041	Peroxiredoxin-6
RAP1B	P61224	Ras-related protein Rap-1b
RBP4	P02753	Retinol-binding protein 4
S100A8	P05109	Protein S100-A8
S100A9	P06702	Protein S100-A9
SAA1	P0DJI8	Serum amyloid A-1 protein
SAA2	P0DJI9	Serum amyloid A-2 protein
SAA4	P35542	Serum amyloid A-4 protein
SDPR	O95810	Serum deprivation-response protein
SERPINA1	P01009	Alpha-1-antitrypsin
SERPINA10	Q9UK55	Protein Z-dependent protease inhibitor
SERPINA3	P01011	Alpha-1-antichymotrypsin
SERPINA4	P29622	Kallistatin
SERPINA5	P05154	Plasma serine protease inhibitor
SERPINA6	P08185	Corticosteroid-binding globulin
SERPINA7	P05543	Thyroxine-binding globulin
SERPINC1	P01008	Antithrombin-III
SERPIND1	P05546	Heparin cofactor 2
SERPINF1	P36955	Pigment epithelium-derived factor
SERPINF2	P08697	Alpha-2-antiplasmin
SERPING1	P05155	Plasma protease C1 inhibitor
SH3BGRL3	Q9H299	SH3 domain-binding glutamic acid-rich-like protein 3
SLC4A1	P02730	Band 3 anion transport protein
SPTA1	P02549	Spectrin alpha chain, erythrocytic 1
TF	P02787	Serotransferrin
THBS1	P07996	Thrombospondin-1
TLN1	Q9Y490	Talin-1
TPM3	P06753	Tropomyosin alpha-3 chain
TPM4	P67936	Tropomyosin alpha-4 chain
TTN	Q8WZ42	Titin
TTR	P02766	Transthyretin
TUBA1C	P0DPH8	Tubulin alpha-1C chain
VCL	P18206	Vinculin
VTN	P04004	Vitronectin
YWHAZ	P63104	14-3-3 protein zeta/delta

Supplemental Data Table 2: Proteins identified in >50% HDL samples

					EU Baseline		EU Weight Gain		SA Baseline		SA Weight Gain	
Gene names	Protein IDs	Protein names	Peptides identified	Sequence coverage	Median LFQ intensity	IQR	Median LFQ intensity	IQR	Median LFQ intensity	IQR	Median LFQ intensity	IQR
ORM1	P02763	Alpha-1-acid glycoprotein 1	7	40.8	8.63E+06	6.30E+06	8.27E+06	1.10E+07	1.27E+07	1.79E+07	1.19E+07	1.82E+07
ORM2	P19652	Alpha-1-acid glycoprotein 2	7	35.3	2.29E+06	4.15E+06	2.63E+06	2.82E+06	2.61E+06	2.55E+06	3.26E+06	1.28E+06
SERPINA3	P01011	Alpha-1-antichymotrypsin	9	26.2	1.19E+06	1.27E+06	1.34E+06	1.22E+06	1.96E+06	2.31E+06	2.28E+06	8.34E+05
SERPINA1	P01009	Alpha-1-antitrypsin	30	64.4	7.17E+07	7.47E+07	1.23E+08	1.40E+08	1.32E+08	1.48E+08	1.43E+08	6.42E+07
A1BG	P04217	Alpha-1B-glycoprotein	10	38.8	8.16E+05	1.45E+06	6.30E+05	1.48E+06	1.44E+06	1.63E+06	1.49E+06	1.27E+06
SERPINF2	P08697	Alpha-2-antiplasmin	8	25.5	0.00E+00	4.24E+06	0.00E+00	3.70E+06	3.07E+06	4.96E+06	3.82E+06	4.33E+06
AHSG	P02765	Alpha-2-HS-glycoprotein	9	44.4	1.75E+07	1.54E+07	1.63E+07	1.94E+07	1.79E+07	1.59E+07	3.21E+07	1.95E+07
AGT	P01019	Angiotensinogen	5	6.8	2.32E+06	1.19E+06	2.50E+06	1.90E+06	3.30E+06	2.36E+06	2.79E+06	2.55E+06
APOA1	P02647	Apolipoprotein A-I	44	85.4	2.31E+09	1.68E+09	2.54E+09	1.04E+09	2.29E+09	7.21E+08	2.30E+09	3.50E+08
APOA2	P02652	Apolipoprotein A-II	9	69	9.17E+08	2.84E+09	1.25E+09	1.68E+09	7.17E+08	3.42E+08	1.05E+09	8.35E+08
APOA4	P06727	Apolipoprotein A-IV	26	62.4	1.97E+07	1.48E+07	2.30E+07	2.55E+07	3.41E+07	2.18E+07	3.85E+07	1.76E+07
APOC1	P02654	Apolipoprotein C-I	4	26.5	1.50E+08	3.48E+08	2.01E+08	2.18E+08	2.01E+08	1.61E+08	1.81E+08	1.56E+08
APOC2	P02655	Apolipoprotein C-II	4	49.5	2.66E+07	8.52E+07	5.80E+07	9.65E+07	4.89E+07	8.50E+07	4.28E+07	6.22E+07
APOC3	P02656	Apolipoprotein C-III	4	48.5	9.14E+07	1.54E+08	1.21E+08	6.96E+08	2.92E+08	9.44E+08	8.15E+07	1.69E+08
APOD	P05090	Apolipoprotein D	9	46.6	1.05E+08	1.41E+08	8.96E+07	1.69E+08	1.14E+08	5.76E+07	1.87E+08	9.52E+07
APOE	P02649	Apolipoprotein E	17	59.9	2.48E+07	3.87E+07	3.12E+07	7.30E+07	3.93E+07	1.67E+07	3.95E+07	1.78E+07
APOF	Q13790	Apolipoprotein F	4	22.1	5.19E+06	1.08E+07	3.67E+06	7.08E+06	0.00E+00	4.96E+06	4.96E+06	6.22E+06
APOL1	O14791	Apolipoprotein L1	11	41.2	7.63E+06	7.71E+06	1.09E+07	5.08E+06	1.13E+07	6.74E+06	1.38E+07	6.38E+06
APOM	O95445	Apolipoprotein M	7	55.3	1.99E+07	2.40E+07	1.71E+07	3.30E+07	1.37E+07	9.70E+06	1.87E+07	7.61E+06
APOH	P02749	Beta-2-glycoprotein 1	14	53	4.48E+06	7.86E+06	5.64E+06	7.64E+06	4.60E+06	1.01E+07	4.69E+06	7.98E+06
CA1	P00915	Carbonic anhydrase 1	8	45.6	3.58E+05	6.61E+05	5.50E+04	5.14E+05	3.09E+05	1.24E+06	6.68E+05	7.78E+05
CETP	P11597	Cholesteryl ester transfer protein	7	19.9	0.00E+00	6.46E+05	0.00E+00	5.77E+05	2.93E+05	5.22E+05	4.99E+05	2.32E+05
CLU	P10909	Clusterin	11	24.5	2.36E+06	3.32E+06	2.86E+06	3.73E+06	3.61E+06	2.06E+06	3.54E+06	3.18E+06
C3	P01024	Complement C3	22	17	9.46E+05	1.44E+06	1.15E+06	1.77E+06	1.17E+06	7.50E+05	1.39E+06	1.13E+06
C4B	P0C0L5	Complement C4-B	24	20.1	1.25E+06	1.23E+06	9.07E+05	1.86E+06	1.97E+06	1.10E+06	2.91E+06	1.99E+06
FGB	P02675	Fibrinogen beta chain	5	12.8	8.08E+05	2.75E+06	3.35E+05	3.07E+06	0.00E+00	4.17E+06	0.00E+00	1.31E+06
GSN	P06396	Gelsolin	10	19.6	0.00E+00	7.78E+05	4.17E+05	1.14E+06	8.08E+05	1.38E+06	8.81E+05	5.94E+05
HPR	P00739	Haptoglobin-related protein	18	60.9	3.15E+06	4.41E+06	3.85E+06	2.69E+06	4.98E+06	6.36E+06	5.00E+06	7.03E+06
HBB	P68871	Hemoglobin subunit beta	12	87.8	8.33E+05	1.27E+06	6.57E+05	1.44E+06	7.69E+05	3.08E+07	7.54E+05	7.22E+05
HPX	P02790	Hemopexin	13	48.3	0.00E+00	8.03E+05	0.00E+00	1.56E+06	9.35E+05	1.89E+06	2.50E+06	1.12E+06
SERPIND1	P05546	Heparin cofactor 2	9	25.7	0.00E+00	9.89E+05	0.00E+00	9.78E+05	8.77E+05	1.05E+06	1.09E+06	4.72E+05
IGHA1	P01876	Ig alpha-1 chain C region	8	36	1.49E+06	1.27E+06	9.38E+05	2.01E+06	1.13E+06	1.43E+06	1.95E+06	1.56E+06
IGHG1	P01857	Ig gamma-1 chain C region	10	48.8	8.06E+05	2.55E+06	1.07E+06	1.25E+06	8.45E+05	1.57E+06	2.04E+06	1.94E+06
IGKC	P01834	Ig kappa chain C region	5	79.4	1.33E+06	2.15E+06	9.41E+05	1.76E+06	1.88E+06	1.61E+06	2.27E+06	1.83E+06

cont.					EU Baseline		EU Weight Gain		SA Baseline		SA Weight Gain	
Gene names	Protein IDs	Protein names	Peptides identified	Sequence coverage	Median LFQ intensity	IQR	Median LFQ intensity	IQR	Median LFQ intensity	IQR	Median LFQ intensity	IQR
IGLC1	P0DOY3	Ig lambda-1 chain C regions	4	60.4	0.00E+00	2.33E+06	1.34E+06	2.02E+06	1.71E+06	1.57E+06	2.08E+06	1.98E+06
KNG1	P01042	Kininogen-1	9	17.1	0.00E+00	2.78E+06	1.12E+06	1.88E+06	1.76E+06	2.86E+06	2.45E+06	2.53E+06
LCAT	P04180	Phosphatidylcholine-sterol acyltransferase	8	29.8	3.76E+06	3.85E+06	6.13E+06	3.59E+06	5.00E+06	3.52E+06	6.63E+06	3.03E+06
PLTP	P55058	Phospholipid transfer protein	5	13.6	2.37E+06	2.99E+06	1.65E+06	2.98E+06	1.75E+06	6.91E+05	2.08E+06	2.11E+06
SERPINF1	P36955	Pigment epithelium-derived factor	13	44.7	3.16E+06	2.70E+06	4.05E+06	3.23E+06	4.03E+06	3.40E+06	2.98E+06	2.08E+06
AMBP	P02760	Protein AMBP	9	31	1.58E+06	1.23E+06	1.61E+06	1.83E+06	1.56E+06	2.07E+06	3.00E+06	1.40E+06
RBP4	P02753	Retinol-binding protein 4	7	40.8	1.10E+06	1.67E+06	1.12E+06	2.11E+06	2.65E+06	4.65E+06	2.27E+06	1.50E+06
TF	P02787	Serotransferrin	30	52.7	1.53E+06	3.72E+06	1.46E+06	4.98E+06	3.18E+06	2.72E+06	3.61E+06	3.78E+06
ALB	P02768	Serum Albumin	80	86.4	2.83E+09	2.74E+09	1.97E+09	1.59E+09	2.86E+09	2.40E+09	2.75E+09	1.34E+09
SAA1	P0DJI8	Serum amyloid A-1 protein	9	62.3	7.99E+05	3.03E+06	1.33E+06	1.91E+06	8.03E+05	7.41E+05	1.50E+06	1.93E+06
PON1	P27169	Serum paraoxonase/arylesterase 1	15	68.7	7.72E+07	8.90E+07	5.97E+07	8.83E+07	7.12E+07	3.24E+07	6.29E+07	2.05E+07
PON3	Q15166	Serum paraoxonase/lactonase 3	8	28.8	1.03E+06	1.45E+06	1.08E+06	1.59E+06	7.45E+05	7.98E+05	7.10E+05	5.88E+05
CLEC3B	P05452	Tetranectin	7	46	6.39E+05	1.14E+06	6.07E+05	9.00E+05	4.85E+05	9.10E+05	6.19E+05	7.63E+05
TTR	P02766	Transthyretin	9	65.3	2.90E+06	3.46E+06	2.74E+06	7.17E+06	4.36E+06	5.43E+06	4.51E+06	3.90E+06
GC	P02774	Vitamin D-binding protein	24	57	6.03E+06	1.52E+07	1.15E+07	1.26E+07	8.01E+06	1.34E+07	1.46E+07	9.98E+06
VTN	P04004	Vitronectin	5	13.2	9.09E+05	1.22E+06	1.14E+06	8.33E+05	1.35E+06	8.53E+05	1.36E+06	6.76E+05
AZGP1	P25311	Zinc-alpha-2-glycoprotein	8	33.6	2.61E+06	2.24E+06	3.11E+06	2.59E+06	3.84E+06	2.05E+06	3.41E+06	9.14E+05

Supplementary figures

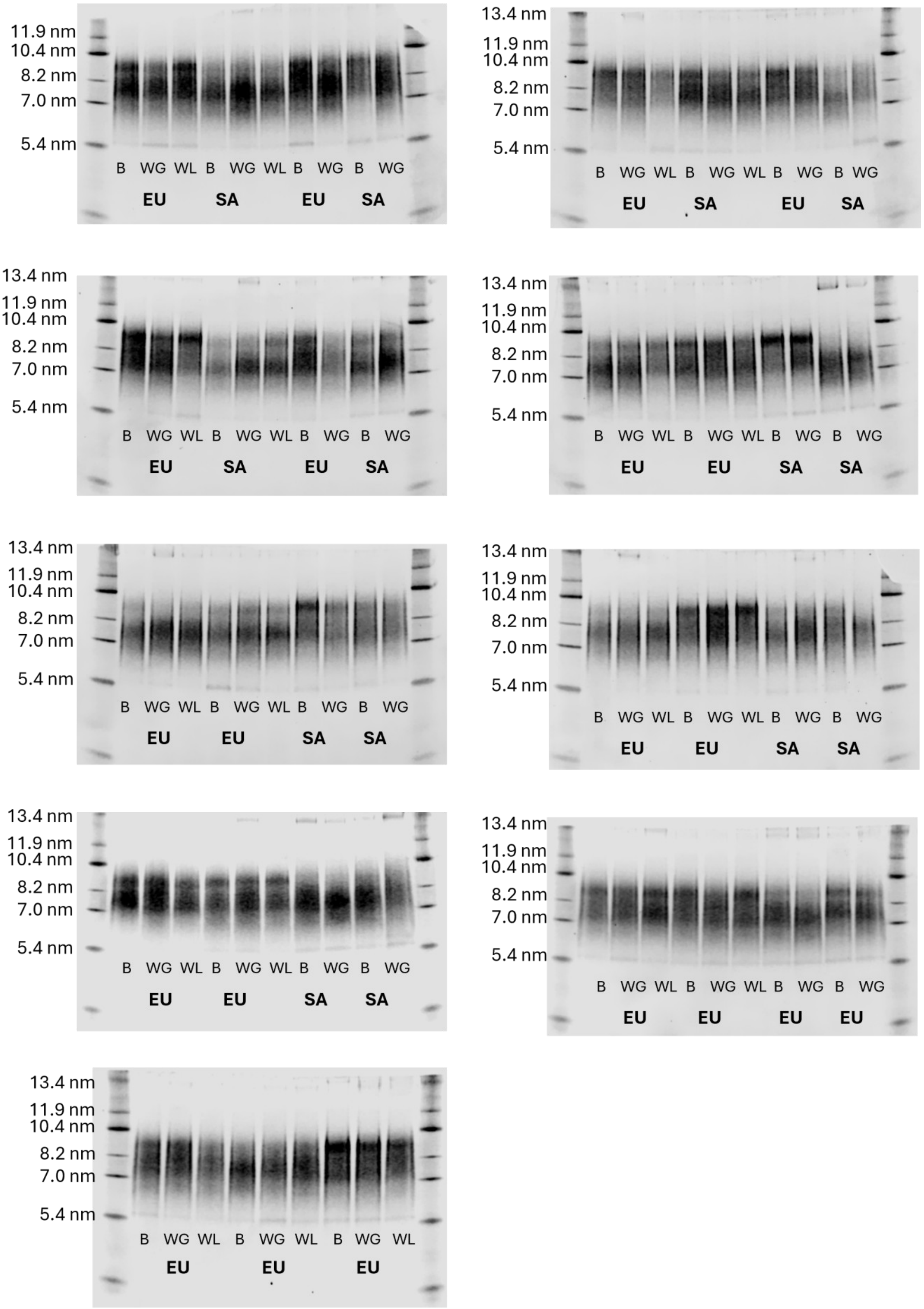

Figure S1 – Coomassie stained electrophoresed white European and South Asian HDL. Gels were imaged using a LICOR Odyssey FC scanner in the 700 nm channel. EU, European, SA, South Asian, B, baseline, WG, weight gain, WL, weight loss.

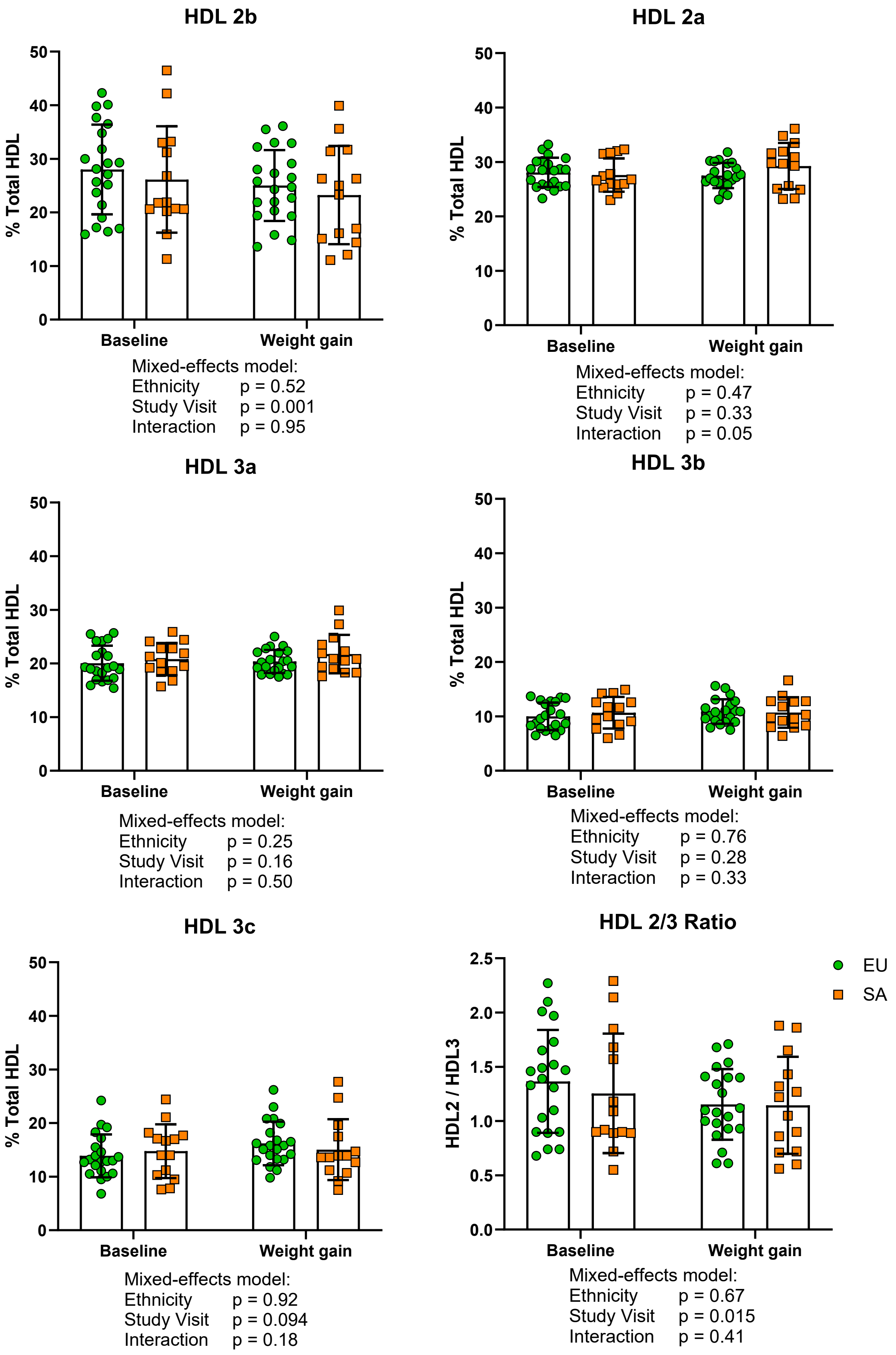

Figure S2 – HDL subclass distribution in white European and South Asian men before and after weight gain. Data expressed as mean ± standard deviation. Data were analysed by mixed effects model and statistical significance assumed at $p < 0.05$. N= 21 Europeans and n=14 South Asians.

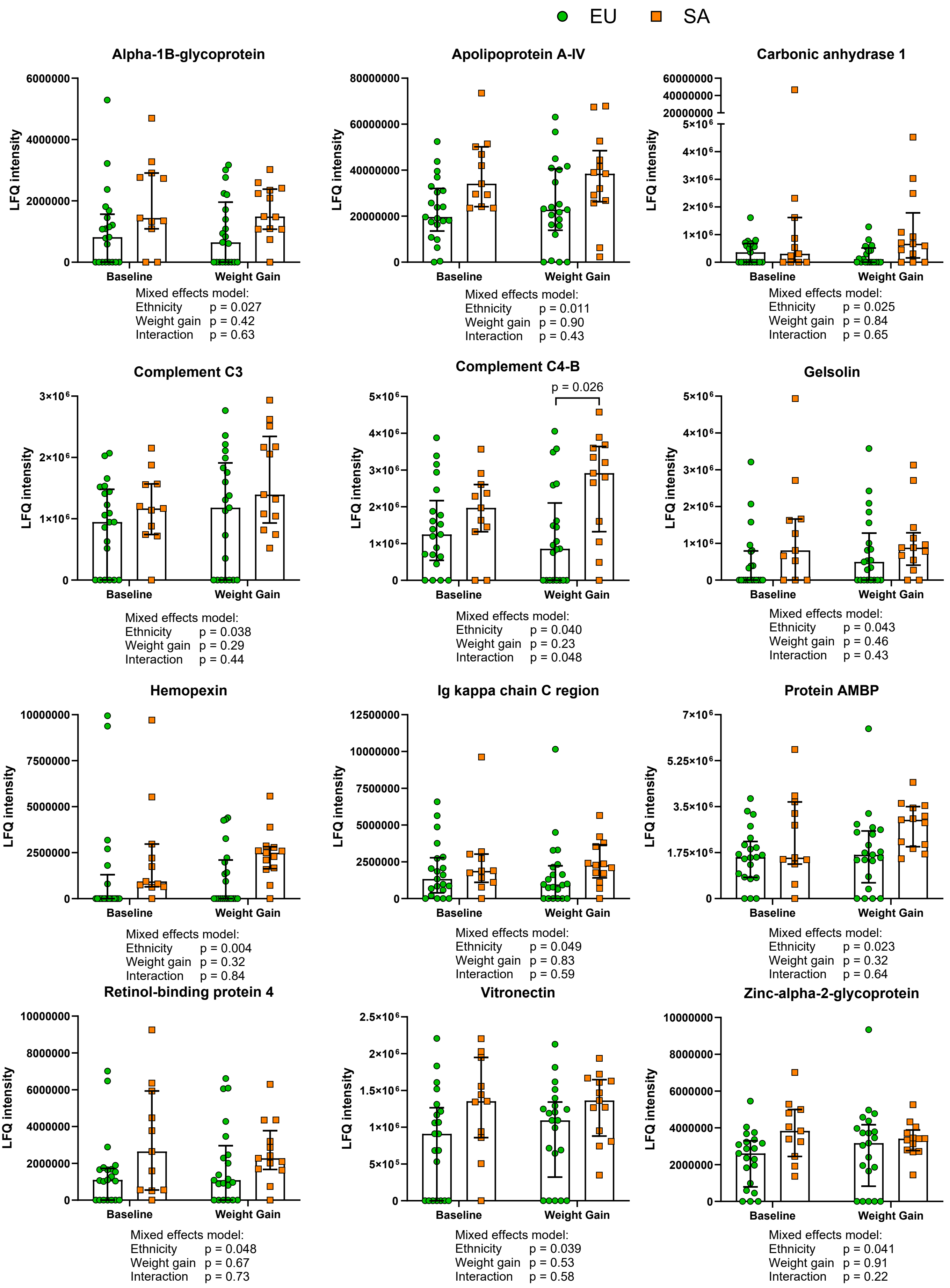

Figure S3 – Individual plots of proteins identified on HDL with significant differences by ethnicity. Data expressed as median ± IQR. Comparisons made by mixed effects model followed by *post hoc* Tukey test. Statistical significance was assumed at $p < 0.05$. LFQ, label-free quantitation. N= 21 Europeans at both baseline and weight gain, n=11 South Asians at baseline and n=13 South Asians with weight gain.

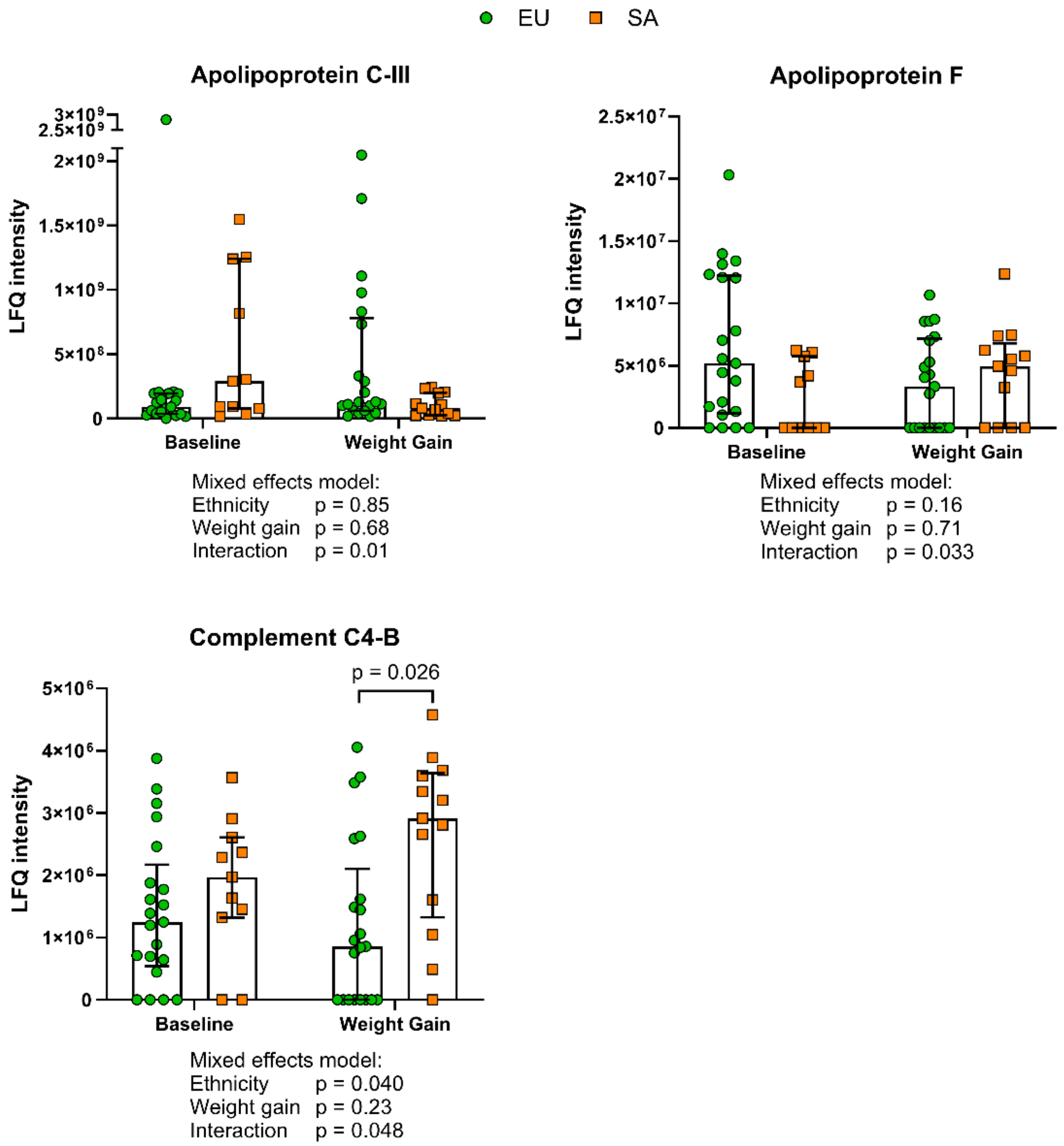

Figure S4 – Individual plots of proteins identified on HDL with significant interactive differences. Data expressed as median ± IQR. Comparisons made by mixed effects model followed by *post hoc* Tukey test. Statistical significance was assumed at $p < 0.05$. LFQ, label-free quantitation. N= 21 Europeans at both baseline and weight gain, n=11 South Asians at baseline and n=13 South Asians with weight gain.
